# 

**Published:** 1861-11

**Authors:** 


### CONTENTS.

ORIGINAL ESSAYS AND COMMUNICATIONS.

Alloys for for Die Metal, Etc. By Dr. B. Wood,........................................
Review of who are Dentists. By J. Allen, D. D. S.,........................................
American Dental Convention. Seventh Annual Meeting........................................

SELECTIONS.

Dentition and its Derangements. By A. Jacobi, M. D.,........................................
Water : its History, Characteristics, Hygienic, and Therapeutic Uses,........................................
Cause which Retard Dental Progress,........................................
Mastication and Articulation of Artificial Dentures,........................................
Convulsions from Dention,........................................
The New Anæsthetic—Keroselene,........................................

EDITORIAL.

Fusible Gauge,........................................ 6[illegible]

Rubber Work,........................................ 6[illegible]

Treatment of Abscess,........................................ 6[illegible]

Hypertrophia of Papilla of the Tongue,........................................ 62[illegible]

The Register,........................................ 6[illegible]

Dental Depot,........................................ 62[illegible]

